# Paternal aging impacts expression and epigenetic markers as early as the first embryonic tissue lineage differentiation

**DOI:** 10.1186/s40246-024-00599-4

**Published:** 2024-03-26

**Authors:** Michelle M. Denomme, Blair R. McCallie, Mary E. Haywood, Jason C. Parks, William B. Schoolcraft, Mandy G. Katz-Jaffe

**Affiliations:** CCRM Genetics, 10290 Ridgegate Circle, Lone Tree, CO 80124 USA

**Keywords:** Advanced paternal age, Blastocyst, Inner cell mass, Trophectoderm, Epigenetics, Methylome, Transcriptome, Offspring health, Neurodevelopmental disorders, Neuronal signaling pathways

## Abstract

**Background:**

Advanced paternal age (APA) is associated with adverse outcomes to offspring health, including increased risk for neurodevelopmental disorders. The aim of this study was to investigate the methylome and transcriptome of the first two early embryonic tissue lineages, the inner cell mass (ICM) and the trophectoderm (TE), from human blastocysts in association with paternal age and disease risk. High quality human blastocysts were donated with patient consent from donor oocyte IVF cycles from either APA (≥ 50 years) or young fathers. Blastocysts were mechanically separated into ICM and TE lineage samples for both methylome and transcriptome analyses.

**Results:**

Significant differential methylation and transcription was observed concurrently in ICM and TE lineages of APA-derived blastocysts compared to those from young fathers. The methylome revealed significant enrichment for neuronal signaling pathways, as well as an association with neurodevelopmental disorders and imprinted genes, largely overlapping within both the ICM and TE lineages. Significant enrichment of neurodevelopmental signaling pathways was also observed for differentially expressed genes, but only in the ICM. In stark contrast, no significant signaling pathways or gene ontology terms were identified in the trophectoderm. Despite normal semen parameters in aged fathers, these significant molecular alterations can adversely contribute to downstream impacts on offspring health, in particular neurodevelopmental disorders like autism spectrum disorder and schizophrenia.

**Conclusions:**

An increased risk for neurodevelopmental disorders is well described in children conceived by aged fathers. Using blastocysts derived from donor oocyte IVF cycles to strategically control for maternal age, our data reveals evidence of methylation dysregulation in both tissue lineages, as well as transcription dysregulation in neurodevelopmental signaling pathways associated with APA fathers. This data also reveals that embryos derived from APA fathers do not appear to be compromised for initial implantation potential with no significant pathway signaling disruption in trophectoderm transcription. Collectively, our work provides insights into the complex molecular mechanisms that occur upon paternal aging during the first lineage differentiation in the preimplantation embryo. Early expression and epigenetic markers of APA-derived preimplantation embryos highlight the susceptibility of the future fetus to adverse health outcomes.

**Supplementary Information:**

The online version contains supplementary material available at 10.1186/s40246-024-00599-4.

## Background

The impact of advanced maternal age (AMA) and the means by which it negatively affects fertility and reproductive outcomes has been well documented, while those of advanced paternal age (APA) remain unclear [1–6]. Nevertheless, an association has been made between paternal age and adverse reproductive outcomes including increased risk of miscarriage [7], and lower birth rates [7, 8], as well as birth defects [9–12], early childhood cancers [13–16], autosomal dominant diseases [17], and neurodevelopmental disorders [18–22]. Apart from genetic mutations, other underlying mechanisms and mode of inheritance for this paternal age effect on offspring health has not yet been fully elucidated, but is hypothesized to be multifactorial in nature. Epigenetic mechanisms that control gene expression, such as DNA methylation, provide one explanation for the non-genetic paternal transmission of offspring disease susceptibility. This study addresses the question of how early in embryo development epigenetic differences and disease risk are identifiable.

Epigenetic regulation involves modifiable and inherited alterations that govern gene expression independently of DNA sequence. A series of crucial epigenetic reprogramming events occur during gametogenesis and preimplantation-stage embryogenesis [23]. In the germline, DNA methylation marks are erased followed by sperm- or oocyte-specific acquisition. A second wave of erasure occurs immediately after fertilization. This demethylation is a rapid and active process in the male pronucleus, but passive and replication-dependent in the female pronucleus. Global remethylation transpires in the blastocyst-stage embryo, initiating tissue-specific methylation. However, imprinted genes and perhaps other developmentally critical regions escape the embryonic portion of epigenetic reprogramming, enabling a mechanism for epigenetic generational inheritance [24]. Unlike genetic errors, the epigenome can be disrupted during these critical periods by various environmental and intrinsic factors, such as parental aging. Indeed, we were the first to report an observed generational inheritance of comparable epigenetic dysregulation in human sperm and blastocysts upon paternal aging, with confirmed significant susceptibility at neurodevelopmental genes [25]. This has led to questioning the subsequent effect on the embryonic transcriptome with respect to disease risk, as well as distinguishing the level of susceptibility of the two embryonic cell lineages of the blastocyst.

The first cell lineage differentiation in the mammalian preimplantation embryo separates the inner cell mass (ICM) and trophectoderm (TE) tissues of the blastocyst. Both acquire lineage-specific DNA methylation that may function to specify the unique transcriptional identities regulating their cell fate. However, the dynamics of the methylome within isolated ICM and TE tissues is not well described, nor has the effect of APA been addressed. Likewise, the influence of altered DNA methylation on ICM and TE transcriptomes is not well understood. Therefore it is important to elucidate both the immediate effects on the expression of genes involved in critical functions during preimplantation development, as well as long-term epigenetic effects on developmental processes and offspring health. Thus, this study was designed to investigate alterations to the APA-related methylome and transcriptome concurrently in both the ICM and TE tissues. Only donor oocyte-derived blastocysts were utilized to strengthen our findings by strategically controlling for confounding effects of advancing maternal age. Here we are the first to demonstrate significant epigenetic dysregulation and gene expression perturbations independently observed as early as the first cell lineage differentiation in association with paternal aging.

## Methods

### Ethics statement

Surplus cryopreserved human blastocysts were donated from couples who had completed IVF treatment, with informed patient consent and Institutional Review Board approval.

### Samples

Blastocysts (*n* = 54) from donor oocyte IVF cycles and normozoospermic patients with advanced paternal age (“APA”; ≥ 50 years) or young paternal age (“young”, ≤ 36 years) were selected to eliminate both known female and male infertility factors. Karyotypically normal blastocysts were scored based on developmental stage, inner cell mass, and trophectoderm appearance [26], and were all morphologically graded as high, transferrable quality (Day 5 or Day 6 of development, grade ≥ 4BB), matched between APA and young for blastocyst grade and timing of development at the time of cyropreservation. Ovarian stimulation, oocyte retrieval, intracytoplasmic sperm injection (ICSI), embryo culture, vitrification, and warming procedures were routinely performed as previously reported [27].

Blastocysts were warmed and mechanically separated into two samples; the entire inner cell mass (ICM), and only trophectoderm (TE) cells. Concurrent DNA and RNA isolations were performed using a modified version of the Dynabeads mRNA DIRECT Micro Kit (Ambion by Life Technologies). Briefly, samples were lysed in Dynabead lysis buffer, then mixed with pre-washed Dynabeads Oligo (dT)_25_ to capture mRNA. The supernatant containing DNA was used for methylome sequencing while the Dynabeads-mRNA complex was washed as the protocol directed, eluted in cold Tris-HCl, and used for transcriptome sequencing, as outlined below.

### Methylome sequencing

Paired ICM and TE blastocyst samples (*n* = 24) were processed and analyzed using the ultra-low DNA input whole genome bisulfite sequencing (WGBS) prep workflow (Zymo Research, Irvine, CA). Samples were prepared for WGBS using an adapted library preparation protocol [28]. Approximately 0.5 pg of methylation sequencing in situ spike-in control oligos were added to help validate and assess the overall workflow and resulting data output. Samples were first bisulfite converted using the EZ DNA Methylation-Direct Kit (Zymo Research) according to the manufacturer’s instructions. This was followed by a second strand synthesis reaction, then Splinted Ligation Adapter Tagging (scSPLAT) using specially designed and pre-annealed oligos. PCR was performed using Illumina TruSeq Unique Dual Indices. Library quality control was performed on the Agilent 4200 TapeStation. Libraries were sequenced on an Illumina NovaSeq 6000 instrument (150 bp PE reads).

Sequence reads from ultra-low input WGBS libraries were identified using standard Illumina base calling software. Raw FASTQ files were adapter and quality trimmed and 15 bases were further trimmed off at the 5’ end of Read 1 according to the Nextera recommendations using TrimGalore 0.6.4. An additional 8 bases were trimmed off at the 5’ end of Read 2, according to the author’s and aligner’s instructions. FastQC 0.11.9 was used to assess the effect of trimming and overall quality distributions of the data. Alignment to the reference genome was performed using Bismark 0.22.3.

Group average methylation values were calculated for all CpGs passing filter. Five million of these for each location (ICM or TE) were sampled at random for the global average methylation density plot. One million were sampled at random for the global average methylation violin plots. Differentially methylated CpGs and differentially methylated regions (DMRs) were detected with Bioconductor dispersion shrinkage for sequencing data (DSS). Significant CpGs and DMRs had FDR ≤ 0.05 and absolute methylation difference ≥ 10%. Each DMR was annotated by overlapping its genomic region with other functional regions including genes, exons, introns, promoters, and CpG islands. Unsupervised hierarchical clustering and cytoband enrichment were generated as previously described [25]. DMR region overlap statistics were calculated using the regioneR package in R and 1,000 permutations.

### Transcriptome sequencing

Paired ICM and TE blastocyst samples (*n* = 30) underwent cDNA conversion and library preparation using the NEBNext Single Cell/Low input RNA library prep kit (Illumina, San Diego, CA), followed by RNA-Seq on the Illumina NextSeq500 with the use of 1 × 76 bp platform. Reads generated were mapped to the human genome (hg19) with the use of gSNAP, and gene expression (values expressed as fragments per kilobase per million [FPKM]) was derived by Cufflinks. Analysis for differentially expressed genes (DEGs) was completed after the removal of technical outliers using DESeq. Due to the limited number of DEGs, a log2FC cutoff was not implemented, but a significance cutoff of FDR < 0.1 was used. For unsupervised hierarchical clustering, significant genes were clustered using Pearson’s rank correlation and average linkage using the “hclust” function with the pheatmap package in R.

### Gene enrichment analysis

Significant overlaps between DMR-associated genes (ICM DMRs, TE DMRs), or differentially expressed genes (ICM DEGs, TE DEGs), and gene lists of interest were calculated using Fisher’s Exact Test followed by p-value adjustment for multiple comparisons using the Benjamini-Hochberg method, with a *p* ≤ 0.05 to define significance. Significant GO and KEGG enrichment was calculated using clusterProfiler in R and significant Reactome enrichment was calculated using ReactomePA. An adjusted *p* ≤ 0.05 was used to define significance.

The current list of known and putative human imprinted genes were downloaded from the Genomic Imprinting Website (http://www.geneimprint.com, accessed 04/03/2023). Autism Spectrum Disorder (ASD) genes were downloaded from SFARI Gene (https://gene.sfari.org/, accessed 04/03/2023), a database of genes implicated in autism susceptibility. Genes were filtered for an enrichment score ≤ 3. Schizophrenia (SZ) genes were downloaded from a SZ GWAS study [29], which identified 287 distinct genomic loci associated with 2091 genes. Bipolar disorder (BD) genes were downloaded from dbBIP, a BD database (http://dbbip.xialab.info/Download, accessed 04/03/2023). Only genes from the Psychiatric Genomics Consortium (PGC) 2 and 3 were used.

### Imprinted methylation validation

Paired ICM and TE blastocyst samples (*n* = 24) underwent targeted bisulfite pyrosequencing for the KCNQ1OT1 imprinting control region (ICR) using the PyroMark Q24 Advanced system (Qiagen). Bisulfite conversion was performed using the EZ DNA Methylation-Direct Kit (Zymo Research), followed by nested PCR amplification using the Platinum II Hot-Start PCR Master Mix (Invitrogen); Pyrosequencing primers were designed in-house with the use of PyroMark Assay Design Software v.2.0.1.15 (Qiagen). Forward primer: GAGTTTATGGTAATGTTTGGTATTTAGAAG, Reverse primer: CGCCAGGGTTTTCCCAGTCACGACCCAAACCACCCACCTAACAAA, universal reverse biotinylated primer in the second round: 5’Biotin-CGCCAGGGTTTTCCCAGTCACGAC, and Sequencing primer: GATGGGAGGTGGGTA. Pyrosequencing reactions were prepared using the PyroMark Q24 Advanced CpG Kit (Qiagen), and the DNA methylation level was calculated as a ratio of the C to T peaks at a given CpG site using PyroMark Q24 Advanced Software v.3.0.0. (Qiagen). Student’s t test was used for methylation differences between young and APA cohorts of ICM or TE, where *p* ≤ 0.05 was considered to be statistically significant.

## Results

### Patient samples

Blastocysts selected for this study were donated with patient and IRB consent from donor oocyte IVF cycles (young fertile women) with normozoospermia based on WHO criteria [30] and internal clinical standards, in order to eliminate contributing female and male factor infertility. Blastocysts were grouped by paternal age, with APA defined as ≥ 50 years, and young fathers as ≤ 36 years selected as controls. Sperm parameters and IVF cycle data are presented in the supplementary (Additional Table 1). Apart from paternal age (mean age ± standard deviation: young: 34.3 ± 1.7 years, APA: 54.4 ± 3.3 years; *p* = 1.18E-14), we did not observe any statistically significant differences between groups in terms of patient variables and sperm parameters, or IVF cycle outcomes including fertilization and blastocyst development rates.

### Methylome analyses

The global ICM and TE paired methylomes from young and APA-derived blastocyst samples were examined using an ultra-low input WGBS approach. An average of 458 million reads and 66% mapping efficiency was achieved, capturing 46 million unique CpGs at 11X coverage and 0.996 correlation coefficient. Despite extremely limited starting DNA input, genomic coverage was high and consistent; gene body (91.6% average coverage), promoters (93.1% average coverage) and CpG islands (94.4% average coverage) (Additional Table 2).

**Fig. 1:**
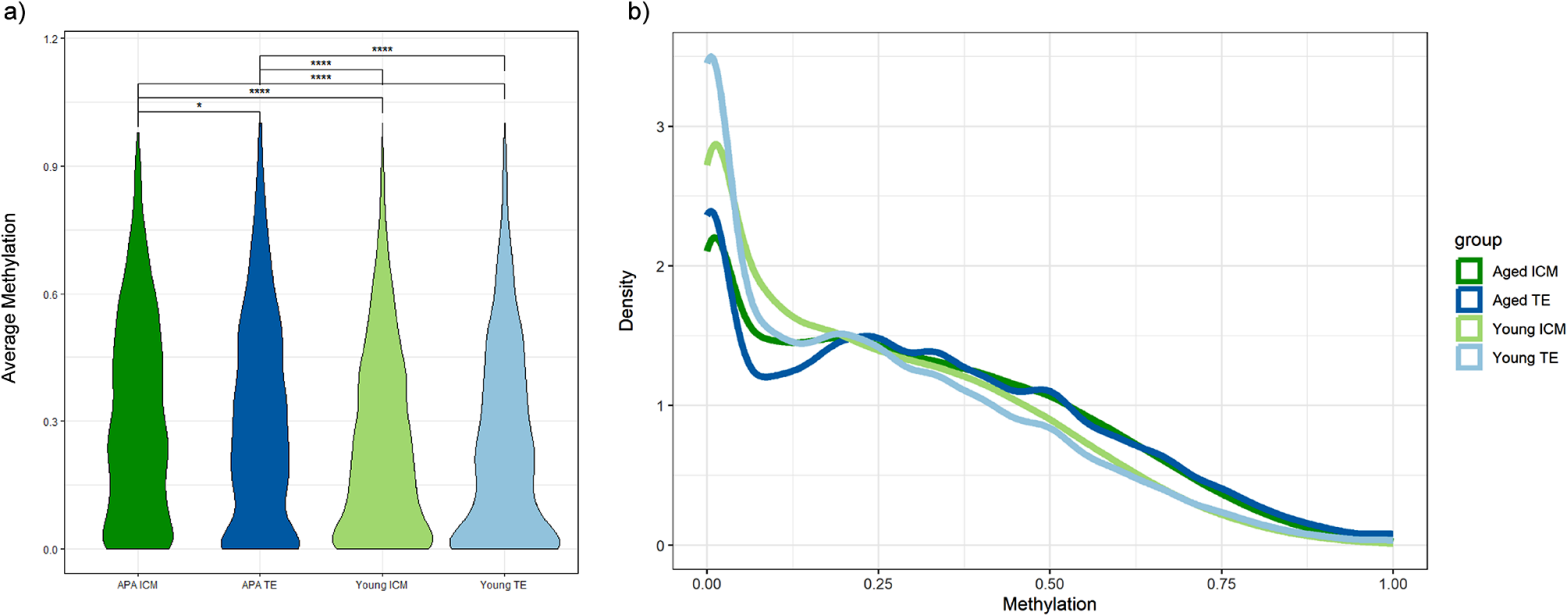
**Global DNA methylation in APA and young blastocysts.** APA is significantly hypermethylated compared to young in both ICM and TE (*p *< 0.05), visualized using (**a**) violin plots and (**b**) line plots

**Fig. 2:**
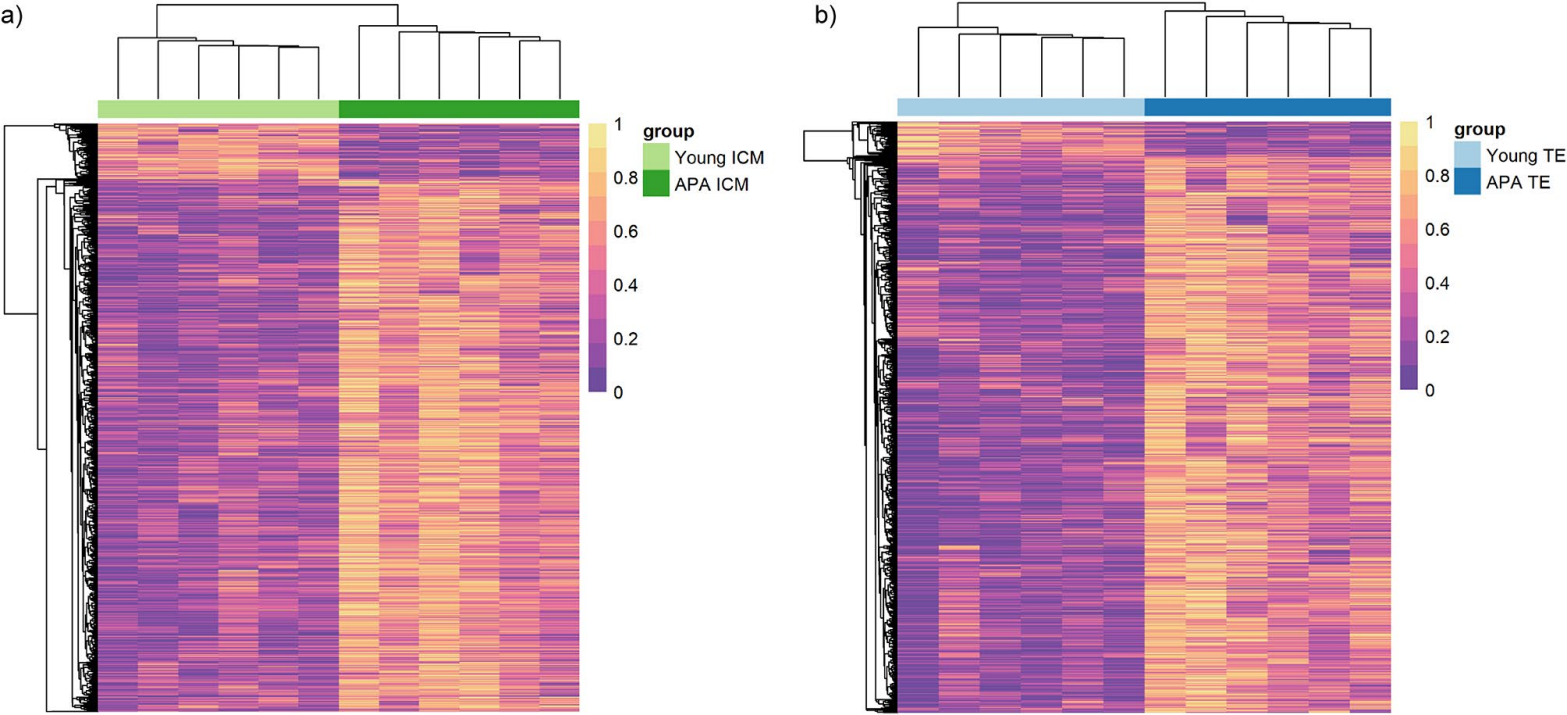
**Significant DMR-associated CpG sites.** Heat map representation of the hierarchical clustering of significant (*p *< 0.05) DMR-associated CpG sites in (**a**) ICM and (**b**) TE, from hypermethylation (yellow; 100%) to hypomethylation (purple; 0%). Samples in both ICM and TE cluster into two distinct groups by young and APA samples

We identified 8,707 and 14,953 CpGs as statistically significant between the young and APA groups for ICM and TE, respectively (*p* ≤ 0.05). A significant increase in global methylation was observed in both tissue lineages of APA-derived blastocysts (Fig. 1a) with a corresponding high proportion of hypermethylated regions compared to young (Fig. 1b). From these, 1,897 ICM and 2,022 TE differentially methylated regions (DMRs) were identified (Table 1). Unsupervised hierarchical clustering analysis of DMRs differentiated between the young and APA groups in both ICM and TE, demonstrating distinct sample branches and unique methylation patterns (Fig. 2).

**Table 1:** **APA ICM and TE significant differentially methylated regions (DMRs) and pathways**

Young (34.3 ± 1.7 years)	APA Inner Cell Mass (ICM)		APA Trophectoderm (TE)	
APA (54.4 ± 3.3 years)	*n*=1,897 DMRs		*n*=2,022 DMRs	
	Hypermethylated	Hypomethylated	Hypermethylated	Hypomethylated
Total DMRs (*p*≤0.05)	1713	180	1890	124
DMR-associated significant CpGs	7771	936	14,061	892
DMR-associated Genes	1114	130	1299	98
GO terms	93		133	
KEGG pathways	0		25	
Reactome	6		4	

**Fig. 3:**
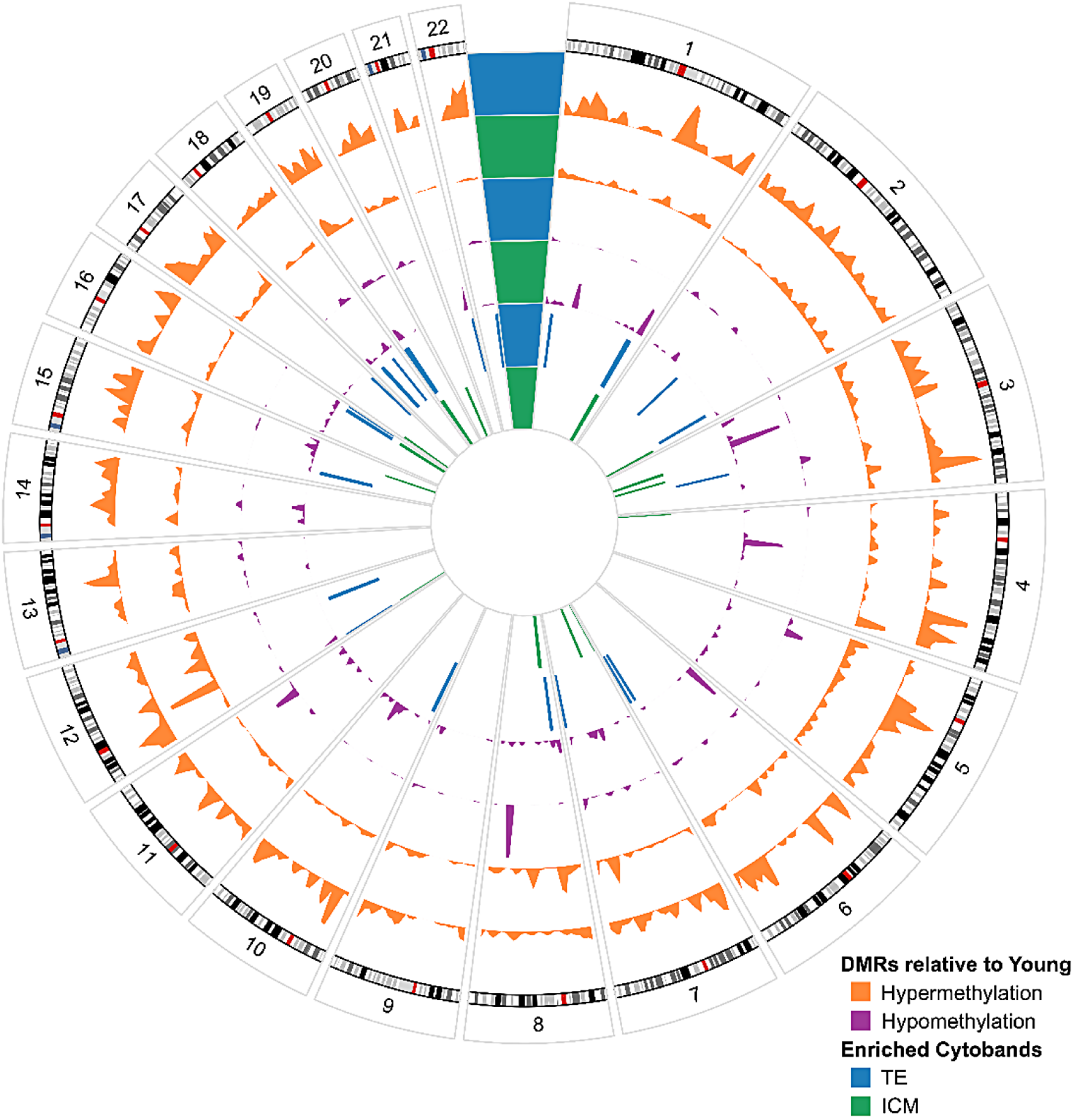
**Genomic circle plot.** Significant cytobands (FDR < 0.05) and DMRs (*p* < 0.05) for APA ICM (green) and TE (blue) throughout the genome. Two innermost layers are significant ICM (green), and TE (blue) cytobands in APA compared to young blastocysts. The two purple layers are hypomethylated (purple) DMRs for ICM and TE, respectively. Finally the outermost layers are hypermethylated (orange) DMRs for ICM and TE, respectively. ChrX and ChrY were excluded from analysis

Individual cytoband enrichment for DMR-associated gene density was analyzed to determine whether specific chromosomal regions were more susceptible to age-related methylation alterations (Fig. 3). We found that methylation alterations appear clustered at certain chromosomal locations, with a significant enrichment identified at 12 cytobands in ICM and 15 cytobands in TE (FDR < 0.05). Five cytobands were independently enriched in both datasets, with the greatest enrichment of these being chr19p13.3 (ICM: q = 3.35E-05; TE: q = 5.83E-03) (Additional Table 3).

**Fig. 4:**
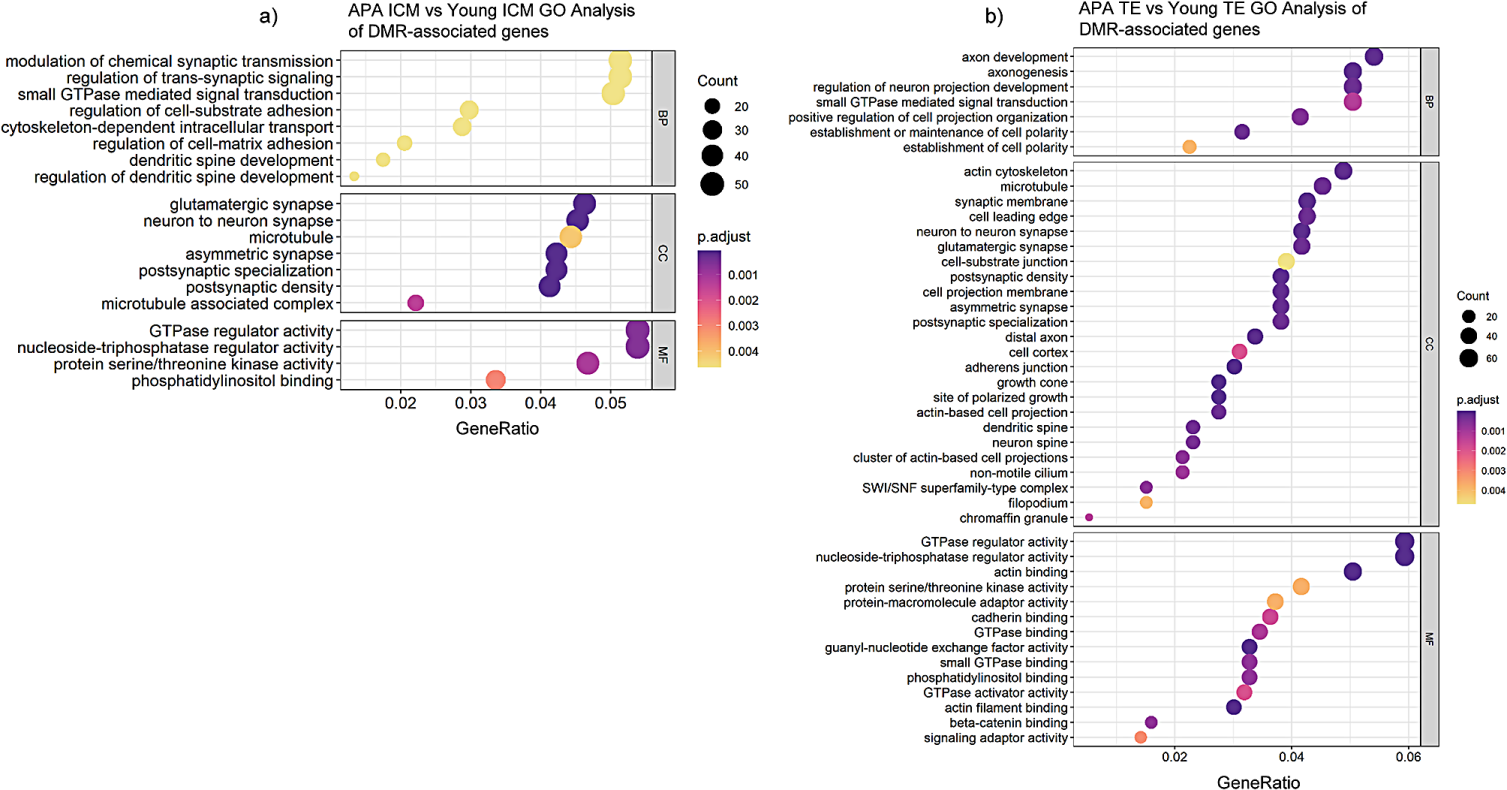
**Gene ontology analysis of DMR-associated genes in ICM and TE.** Significant gene ontology (GO) enrichment analysis (*p* < 0.005) categorized into biological process (BP), cellular component (CC), and molecular function (MF) groups for (**a**) ICM (n=19 GO) and (**b**) TE (n=45 GO) in APA compared to Young blastocysts.

We identified 1,223 and 1,383 DMR-associated genes in ICM and TE, respectively (*p* ≤ 0.05), with 251 genes overlapping both datasets (*p* = 1.46E-102). To gain functional insight into the genes with APA-induced altered DNA methylation, we performed a gene ontology enrichment analysis on the significant DMR-associated genes in the ICM (*n* = 93) and TE (*n* = 131). Interestingly, both ICM and TE DMRs were highly enriched for genes involved in neuronal signaling and synaptic transmission, as well as genes involved in regulation of embryonic development and cell differentiation and proliferation including GTPase regulator activity and protein kinase activity (Fig. 4), suggesting an overall vulnerability for embryonic and neurodevelopmental pathways in association with paternal aging. Many of the gene ontology terms were identical between the two comparisons, especially within the cellular component and molecular function categories. Approximately one-quarter of genes within these identical GO terms overlapped (27% in ICM, 19% in TE, *p* < 0.05), suggesting that similar regions of the genome in both lineages are susceptible to disruption by paternal aging. Reactome enrichment identified similar processes within the ICM including neuronal system and various signal transduction processes, which are involved in regulation of cellular events including migration, adhesion, division, establishment of cellular polarity and intracellular transport during embryogenesis. Similar signal transduction processes were identified in APA-derived TE DMRs, as well as hormone-driven regulatory pathways involved in energy metabolism (Additional Fig. 1). We performed a statistical comparison of our methylome results to previous studies on paternal age-related human sperm and blastocyst methylation changes [25, 31, 32]. Significant overlap of DMR-associated genes was identified for both the APA-derived ICM and TE tissue lineages, demonstrating generational correlation of an altered methylation landscape in sperm and embryo (Additional Table 4). As several neuronal signaling pathways were highly represented among regions of the genome that were differentially methylated, and APA is known to be associated with increased risk to neurodevelopmental disorders, we next compared publicly available gene lists to our DMR-associated genes (Table 2). For APA-derived ICM, a significant enrichment was identified for genes implicated in autism spectrum disorder (149 genes, q = 6.9E-41; OR: 4.3) and schizophrenia (108 genes, q = 1.4E-03; OR: 1.4). Likewise, for APA-derived TE, significant enrichment was identified for autism spectrum disorder (150 genes, q = 6.6E-36; OR: 3.8), schizophrenia (122 genes, q = 7.3E-04; OR: 1.4), and bipolar disorder (30 genes, q = 9.1E-03; OR: 1.7). Again approximately one-quarter of genes implicated in neurodevelopmental disorders are present in both datasets (25% in ICM, 24% in TE, *p* < 0.05).

**Table 2:** **Neurodevelopmental disorder associations for differentially methylated regions (DMRs)**

Disease	Overlapping genes	Fold enrichment (odds ratio)	OR 95% confidence interval	*P*-Value	FDR
**APA Inner Cell Mass (ICM)**					
Autism spectrum disorder	149/1127	4.30	3.56-5.18	1.0E-41*	1.0E-40*
Schizophrenia	108/2092	1.43	1.16-1.76	8.5E-04*	1.9E-03*
Bipolar disorder	21/430	1.32	0.81-2.05	0.20	0.24
**APA Trophectoderm (TE)**					
Autism spectrum disorder	150/1127	3.77	3.12-4.53	3.6E-36*	3.1E-35*
Schizophrenia	122/2092	1.44	1.17-1.74	3.2E-04*	7.8E-04*
Bipolar disorder	30/430	1.71	1.13-2.4	7.6E-03*	1.2E-02*

* Indicates significance FDR≤0.05

Finally, given that imprinted genes function in the developing brain and exhibit various characteristics similar to neurodevelopmental disorders, we chose to compare the current list of known and putative human imprinted genes to the DMR-associated genes identified between the young and APA groups for ICM and TE. We found that ICM DMRs overlapped with 18 imprinted genes (q = 6.0E-03; OR: 2.2) and 3 imprinting control regions (*MEG3, SNRPN, KCNQ1OT1*) while TE DMRs overlapped with 20 imprinted genes (q = 3.9E-03; OR: 2.1) and 3 imprinting control regions (*MEG3, PLAGL1, N4BP2L1*) (Additional Tables 5 and 6). The KCNQ1OT1 imprinting control region (ICR) was selected for targeted methylation validation in an additional cohort of young and APA blastocysts. Hypermethylation was identified with statistical significance found in the ICM samples (Young ICM: 39.3%; APA ICM: 47.8%; *p* = 0.029, Young TE: 41.0%, APA TE: 47.2%, p = ns), suggesting an aberrant gain in methylation from the unmethylated paternal contribution (Additional Fig. 2).

### Transcriptome analyses

**Fig. 5:**
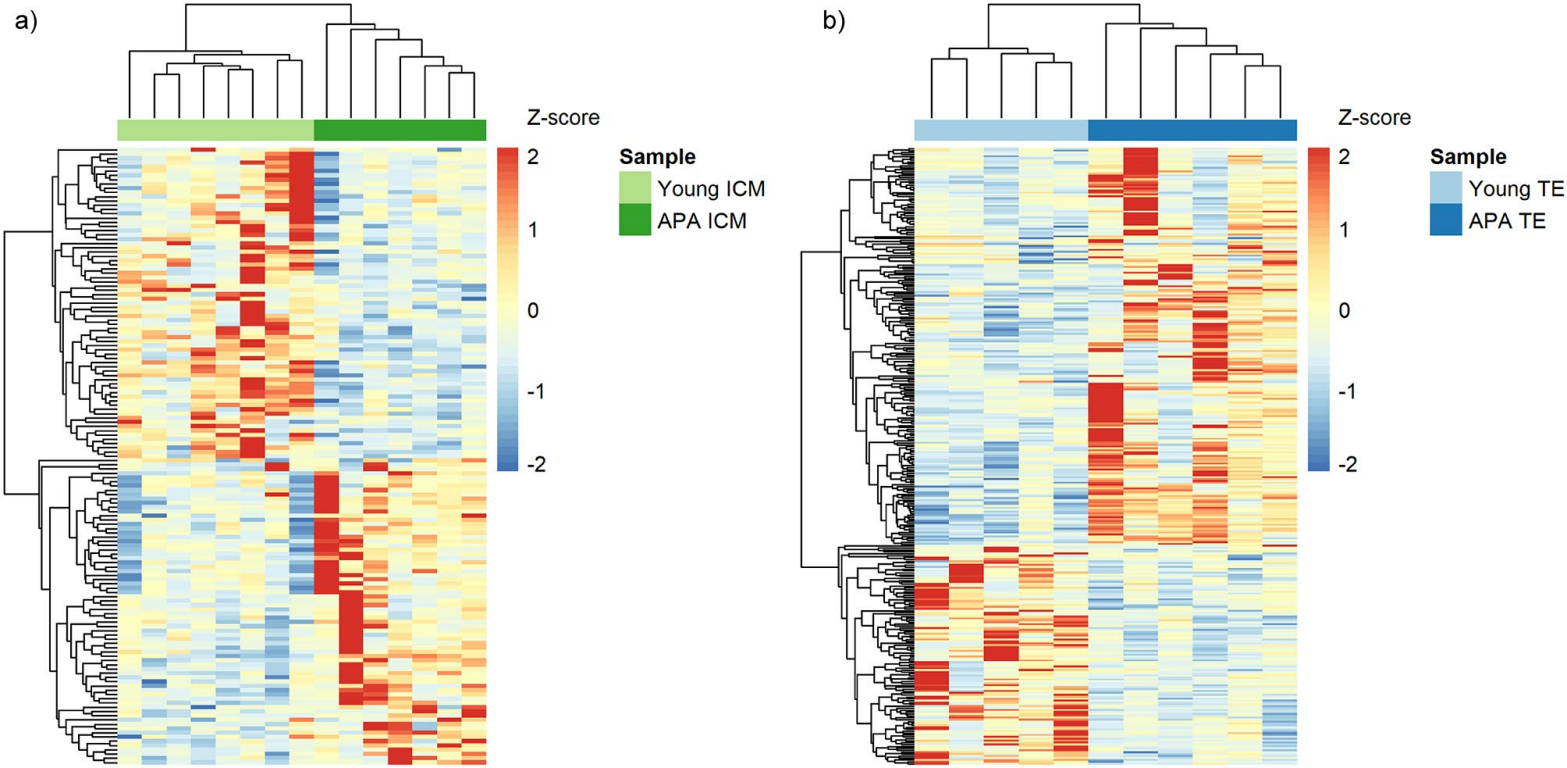
**Significant differentially expressed genes.** Heat map representation of the hierarchical clustering of significant (q < 0.1) differentially expressed genes (DEGs) in (**a**) ICM (n = 145 DEGs) and (**b**) TE (n = 323 DEGs), where a positive z-score (red) corresponds to up-regulation and a negative z-score (blue) corresponds to down-regulation relative to the mean. Samples in both ICM and TE cluster into two distinct groups by young and APA samples

A low input mRNA-seq approach was used to examine the paired ICM and TE transcriptomes from the same young and APA-derived blastocysts. The filtered reads distribution averaged 51 million single-end reads with 73% uniquely aligned (Additional Table 7). Altogether, 31,497 transcripts were expressed in combined ICM and TE samples. Statistical analysis was performed to identify differentially expressed genes (DEGs). Controlling for cellular location, we identified 145 genes in ICM, and 323 genes in TE with significant differential expression in blastocysts derived from aged fathers relative to those derived from young fathers (FDR < 0.1) (Table 3). Within the APA-derived ICM, 72 genes had increased gene expression and 73 had decreased gene expression, while those within the APA-derived TE had 208 increased and 115 decreased, compared to those derived from young fathers. Unsupervised hierarchical clustering analysis of significant DEGs differentiated between the young and APA in both ICM and TE tissue groups, demonstrating distinct sample branches and unique expression patterns (Fig. 5).

**Table 3:** APA ICM and TE significant differentially expressed genes (DEGs) and pathways

Young (34.3 ± 1.7 years)	APA Inner Cell Mass (ICM)		APA Trophectoderm (TE)	
APA (54.4 ± 3.3 years)	*n*=145 DEGs		*n*=323 DEGs	
	Upregulated	Downregulated	Upregulated	Downregulated
Total DEGs (FDR≤0.1)	72	73	208	115
GO terms	18		0	
KEGG pathways	10		0	

For functional perspective, we performed pathway analysis and gene ontology enrichment analysis for differentially expressed genes in APA-derived ICM and TE portions. Significant neuronal signaling pathways were identified within the ICM of APA blastocysts, including GABAergic synapse and glutamergic synapse (FDR ≤ 0.1; Additional Fig. 3a), with all top ten pathways having some involvement in neurodevelopmental signaling and many involved in the etiology of autism spectrum disorder. Gene ontology enrichment analysis for affected biological processes include biological regulation of hormones and peptides (transport and secretion), while the molecular function category included organic acid and amino acid localization (transport and secretion) (FDR ≤ 0.1; Additional Fig. 3b). Linear regression analysis illustrated a significant progressive increase in gene expression as paternal age increased for ten genes within the ICM (Additional Fig. 4). In stark contrast, zero significant pathways or gene ontology terms were identified for the differentially expressed genes in the TE of APA-derived blastocysts relative to younger fathers.

Genes implicated in neurodevelopmental disorders such as autism spectrum disorder, schizophrenia and bipolar disorder, were not significantly enriched among the differentially expressed genes of the ICM or TE upon paternal aging. Combining results from all three disorder datasets, only 12 genes were identified with altered expression in the APA-derived ICM, and 28 in the APA-derived TE (Table 4). Likewise, there was no statistical enrichment for human imprinted genes among the transcriptome datasets, with only two imprinted genes differentially expressed in the ICM and one imprinted gene in the TE of APA blastocysts (Additional Table 5).

**Table 4:** **Neurodevelopmental disorder associations for differentially expressed genes (DEGs)**

Disease	Overlapping genes	Fold enrichment (odds ratio)	OR 95% confidence interval	*P*-Value	FDR
**APA Inner Cell Mass (ICM)**					
Autism spectrum disorder	4/1127	0.77	0.21-2.01	0.82	0.82
Schizophrenia	6/2092	0.73	0.22-1.36	0.31	0.73
Bipolar disorder	2/430	1.01	0.12-3.72	0.73	0.82
**APA Trophectoderm (TE)**					
Autism spectrum disorder	8/1127	0.68	0.29-1.37	0.36	0.73
Schizophrenia	18/2092	0.83	0.49-1.34	0.50	0.80
Bipolar disorder	2/430	0.45	0.05-1.65	0.34	0.73

* Indicates significance FDR≤0.05

### Methylome and transcriptome overlap

There is very limited overlap with no correlation between DMR-associated genes in the methylome data and the differentially expressed genes in the transcriptome data for either the ICM or the TE upon paternal aging. Only five overlapping genes exist in the APA-derived ICM between significant DMR-associated genes and significant RNA-seq differentially expressed genes (ADAMTS20, DOP1B, GDPD4, NOS2, SPAG16). None of these five are implicated in neurodevelopmental disorders, nor are present in any of the methylome gene ontology terms. Likewise, only eleven overlapping genes exist in the APA TE between significant DMR-associated genes and significant RNA-seq differentially expressed genes (APOM, ARL17B, COL20A1, FAM53A, MIR646HG, OACYLP, OSBPL1A, PRKAG2, TPGS2, TRIM62, URGCP). ARL17B is implicated in schizophrenia, TPGS2 is found in the microtubule gene ontology term, and PRKAG2 is part of 4 pathways (AMPK signaling pathway, Lysine degradation, Cholinergic synapse, GnRH secretion) as well as the protein serine/threonine kinase activity gene ontology term. Finally, zero overlapping pathways exist, as APA-derived TE transcriptome did not reveal any significant pathways or gene ontology terms.

Nevertheless, both datasets harbor significant genes involved in neuronal signaling. In fact, glutamergic synapse, the main excitatory neurotransmitter in the brain, is identified in both the ICM transcriptome and the ICM and TE methylomes, and presents a possible route for identifying early markers to neurodevelopmental disease in blastocysts generated by aged fathers.

## Discussion

A greater health risk has been identified for children conceived by fathers over 50 years of age, including an increased adjusted odds ratio (OR) for developing the neurodevelopmental disorders autism spectrum disorder (OR: 2.26 to 3.37), schizophrenia (OR 1.59–4.62), and bipolar disorder (OR 1.27–2.84) [33]. While the paternal age effect appears to be a multifaceted phenomenon, the association with these multifactorial disorders is robust and reproducible. Paternal factors, such as epimutations arising in the sperm as men age, may occur at genes essential for embryonic development or implicated in offspring health conditions, providing a potential mechanism for these adverse outcomes. In particular, there have been studies demonstrating DNA methylation errors at genes important for neurodevelopment in sperm upon advanced paternal age [25, 31, 32, 34]. Yet, minimal information exists on the possible consequences that age-related sperm methylation errors may have post-fertilization on embryogenesis and offspring health. In a mouse study that analyzed DNA methylation in aged sperm, similar epigenetic errors were observed in offspring embryonic brain as well as effects on offspring behavior [35]. Likewise, our group reported generational inheritance of corresponding epigenetic dysregulation in sperm and embryo during IVF treatment [25]. Here, we are the first to dissect the epigenomes of the ICM and TE cell lineages of human blastocysts in response to paternal aging. We are also the first to describe an altered transcriptome that appears to be independent of the DNA methylation changes, yet still impacting gene pathways implicated in neuronal signaling and neurodevelopmental disorders, specifically in the inner cell mass.

Advanced paternal age has the potential to lead to a detrimental effect on semen quality as well as increased DNA fragmentation, particularly in men over 50 years of age [36]. We purposefully excluded patients that were outside normal clinical semen parameter ranges [30], to eliminate any possible confounding influence from male factor infertility. Studies on clinical reproductive outcomes of older men are oftentimes difficult to interpret due to the lack of adjustment for maternal age. For our study we strategically included only donor oocyte IVF cycles (young fertile women) to control for the profound effects of female aging on fertility. In spite of our study design, the blastocyst epigenome and transcriptome were both disrupted in these embryos derived from advanced paternal age compared to young paternal age counterparts.

A significant increase in global methylation was observed in both ICM and TE lineages of APA-derived blastocysts with a corresponding high proportion of hypermethylated regions. These DMRs were clustered at significant cytobands throughout the genome; four cytobands were independently enriched in both ICM and TE tissues, with the greatest enrichment being chr19p13.3. We previously identified this subtelomeric region as being enriched in sperm and intact blastocysts derived from APA fathers [25]. Subtelomeric regions may be excluded from large-scale epigenetic reprogramming events [37–39], or a looser chromatin conformation may be required to access the large number of genes in this region. Both explanations support an avenue of vulnerability to epigenetic disruption.

A statistically significant overlap of DMR-associated genes was detected among ICM and TE datasets from aged fathers compared to young. With that said, very similar gene ontology enrichment was observed, particularly in the cellular compartment and molecular function categories. Many overlapping cellular component terms centered on neurotransmission activity, such as glutamatergic synapse, neuron to neuron synapse, asymmetric synapse, postsynaptic specialization, and postsynaptic density. We observed a similar association with neurotransmission, specifically opioid signaling, in our prior work on APA-derived sperm and blastocysts [25]. Molecular function overlapping terms included various activities required for embryonic development like regulation of cell proliferation and differentiation, metabolism, and cell signaling. Such terms included GTPase regulator activity, NTPase regulator activity, protein serine/threonine kinase activity, and phosphatidylinositol binding. Finally, while the biological process category was mostly unique between ICM and TE, the premise of the terms were similar and relevant to the other categories; neurotransmission and embryonic developmental activity. Reactome enrichment identified comparable pathways in the ICM of APA-derived blastocysts, including neuronal system and various signal transduction processes. For example, RHO GTPases are involved in the regulation of many fundamental cellular processes important during embryonic development, including morphogenesis, polarity, movement, and cell division. As a consequence, RHO GTPases play important roles in neuronal development, and disruption plays a role in the etiology of neurodegenerative diseases [40]. Similar signal transduction processes were identified in APA-derived TE DMRs, as well as hormone-driven regulatory pathways involved in energy metabolism.

APA-derived ICM and TE DMRs encompassed statistically significant enrichment for neurodevelopmental genes implicated in autism spectrum disorder and schizophrenia. The incidence of these neuropsychiatric conditions is known to increase progressively with increasing paternal age [33]. Evidence for abnormal DNA methylation in association with these disorders is also described [41–43]. Our group reported a relationship between paternal aging and epimutations at genes implicated in these disorders in sperm and intact blastocysts [25], with 108 genes overlapping our present study in ICM and TE tissues. A number of these genes were also present in pathways identified by gene ontology enrichment analysis, such as ANKS1B, CACNA1C, IGSF9B and SHANK3. Mechanistically, alterations to DNA methylation may occur in sperm over time as men age, and persist through fertilization at this group of genes which appear to be highly susceptible to epimutations.

Another group of genes that present a level of vulnerability are imprinted genes, since gametes acquire parent-of-origin specific genomic imprints that then persist through fertilization and embryonic development by evading epigenetic reprogramming [44]. Imprinted genes are known to play an essential role in brain development and contribute to some neurodevelopmental conditions [45], as well as development of the placenta. Upon paternal aging in a mouse model, methylation differences were found in brain-expressed imprinted loci, with concurrent behavioral changes [46]. Twenty-three imprinted genes were found to be altered within the ICM or TE of APA-derived blastocysts compared to young, with seven altered in both lineages. In particular, DLGAP2 is one imprinted gene that had differential methylation in both the ICM and TE, as well as in the intact blastocyst and sperm from aged fathers [25]. This gene is also found in the overlapping gene ontology terms involved in neurotransmission from ICM and TE DMRs, and has been implicated in the development of autism [43, 47, 48]. Imprinting control regions (ICRs) were also impacted within the ICM and TE of APA-derived blastocysts. Results from our targeted methylation analysis at the KCNQ1OT1 ICR validates the hypermethylation observed for this region in the global methylome data, as well as aligns with a paternal age effect representing an aberrant gain of methylation presumably from the sperm contribution. To translate these results to later stages in embryonic development, a comparable gain of methylation was observed at the Kcnq1ot1 ICR in mouse embryonic placentas derived from aged males [49], and older paternal ages have been linked to hypermethylation in human IVF placentas collected at time of delivery [50].

There was a remarkably large overlap of DMR-associated genes between the ICM and TE methylomes upon paternal aging, suggesting that many of the same genomic regions may be susceptible to methylation dysregulation. Concordant with the increased risk of impaired neurocognitive phenotypes observed in offspring, paternal age-induced epigenetic alterations occurred at genes involved in several neuronal signaling gene ontology terms, imprinted genes, and genes implicated in neurodevelopmental disorders. While this result was mostly expected in the ICM methylome, we were surprised to discover this to also be true in the TE methylome. Interestingly, a number of recent studies have discussed the placenta-brain-axis (PBA), such that abnormal regulation of certain genes in the placenta affect the fetal brain [51–55]. The placenta produces neurotransmitters that may circulate and influence brain development, and it has been implicated that neurobehavioral disorders such as autism spectrum disorder likely trace their origins back to placental disturbances. Due to this intimate relationship, it has been proposed that the placenta is a promising tissue for identifying DNA methylation changes at genes that also function in the fetal brain, with possible associations to autism spectrum disorder diagnoses.

Like the methylome, paternal age strongly impacted ICM and TE transcription. However, only differentially expressed genes in the ICM of APA-derived blastocysts exhibited statistical enrichment for gene ontology terms and pathways. Neurotransmission and other signaling pathways known to play a role in the brain, such as glutamergic synapse, GABAergic synapse, apelin signaling and relaxin signaling, were identified. Significantly altered genes associated with some of these pathways include SLC38A3 and ADCY5, which are critical for amino acid and amide transport vital to not only ICM proliferation, but also future brain function [56, 57]. Others include NOS2, an important signaling molecule of the central nervous system associated with neurotransmission and diverse brain disorders [58], and AGTR1, which is associated with susceptibility to brain neurodegeneration [59]. Linear regression analysis illustrated a progressive increase in gene expression as paternal age increased for eleven genes, two of which show an association with brain function; ITGA2 has been linked to axonogenesis [60] while SLC25A27 is involved metabolism in the brain, and is implicated in autism spectrum disorder as well as a possible connection to schizophrenia [61, 62]. Interestingly, all of the top ten KEGG pathways have some involvement in neurodevelopmental signaling and many are involved in the etiology of autism spectrum disorder. Numerous key gene ontology terms were identified including the regulation of hormones and peptides through transport and secretion, as well as amino acid and organic acid transport activity. Additional DEGs in the ICM of APA-derived blastocysts have been determined critical for blastocyst cell proliferation, such as IL6 involved in ICM cell numbers and expansion. Yet, despite this strong association with pathways important to brain function, it may be too early in development to impact transcription for genes implicated in neurodevelopmental disorders or imprinted genes, as none of these experienced a statistical enrichment among the differentially expressed genes in either the ICM or TE of blastocysts derived from APA fathers.

Though both the ICM and TE comparisons lead to a similar number of differentially expressed genes, by comparison we found no significant signaling pathways or gene ontology terms enriched in the trophectoderm of aged fathers. As the trophectoderm lineage is responsible for implantation into the uterus and forms the placenta, our results support clinical data showing comparable IVF outcomes of donor oocyte APA cycles to those of younger men [5, 63]. Rather, the ICM lineage that gives rise to the fetus displayed various altered pathways and gene ontology terms important in brain function, which may contribute to downstream consequences of offspring health and align with clinical reports of greater risks after birth.

Interestingly, enrichment for the glutamatergic synapse pathway was identified in both the APA-derived methylome and transcriptome datasets. Glutamatergic synapse is involved in establishing neuronal network connections during brain development and mediating the cellular processes required for neurotransmission [64]. Thus, disruptions may play a relevant role in neurocognitive disease, and presents a possible route for identifying early markers to neurodevelopmental disorders.

Mechanisms exist for aged sperm to transfer altered chromatin signatures to the embryo. However, it is unclear when the adverse effects on offspring health initially occur. The question remains, how early in embryo development epigenetic differences and disease risk are identifiable. Here, we report alterations to the methylome and transcriptome as early as the blastocyst stage, with differences observed in the first two cell lineages. However, despite these events taking place within the same embryos, there is very limited overlap and no correlation between DMR-associated genes in the methylome data and the differentially expressed genes in the transcriptome data for either the ICM or the TE lineages. This suggests that two independent mechanisms are likely at play during embryogenesis; the immediate cascade of altered gene expression occurring in the APA-derived preimplantation embryo, and the long-term alteration of epigenetic marks potentially inherited from APA sperm that will influence transcription later in development and after birth. Since altered DNA methylation does not appear to directly influence gene expression at the preimplantation stage, it may be that additional epigenetic mechanisms, such as histone modifications and miRNA expression profiles, or another paternal effect factor, is leading to the immediate and transient changes in blastocyst gene expression. The large number of synthesized mRNA transcripts expressed in the preimplantation embryo likely represent the plasticity of the embryo and its ability to adapt to its ever-changing environment. Excess transcripts present in the blastocyst may be generated only for utilization if needed, with post-transcriptional regulatory processes occurring at this time. Thus, the transcriptome represents a snapshot of the present timepoint, and is likely not a true reflection of future health and disease risk. Nevertheless, it was interesting to observe transcriptomic dysregulation involving numerous neuronal signaling pathways in the ICM only, showing potential early disruption to future brain development. Meanwhile, methylome changes are stable and inherited as cell division and differentiation occur during development, and are likely the mechanism leading to future predisposition to disease. Both ICM and TE methylomes were highly impacted at genes implicated in neurodevelopmental disorders and neuronal signaling pathways, with possible subsequent transcriptional changes occurring later in development.

It is important to highlight the strengths and limitations to this study. The use of only donor-oocyte IVF cycles controlled for the profound influence of female aging on fertility and was a strategic approach to isolating the effects of paternal age on embryos. Significant patient confounders such as paternal BMI, smoking status, semen analyses results and IVF cycle outcomes were controlled for, however, additional medical history like familial backgrounds and offspring health conditions are not collected by the IVF clinic. The small sample sizes used for genome-wide methylome and transcriptome analyses, although necessary for economic purposes, could represent a technical limitation that may have adversely impacted the statistical power of our results. Finally, the starting input material was also considerably limited, especially following the dissection of ICM and TE tissues and further isolation of DNA and RNA from the same embryo. However, the ability to examine methylome and transcriptome within the same embryos and tissues was a significant strength for the aim of our study.

Based on the divergent ages between our young and APA groups, it is difficult to ascertain if a specific paternal age threshold leads to altered methylation and transcription patterns. Epidemiological studies suggest a linear relationship between increasing paternal age and the risk for neurodevelopmental disorders in their offspring [33]. Likewise, we and others have reported a linear relationship between increasing paternal age and methylation alterations in sperm [25, 65]. However, APA is a subtle and varying effect to male reproductive potential, and inter-individual variability between males exceeds age-associated variation [65]. Similarly, not every blastocyst that is derived from an APA father will become a child that presents with a neurodevelopmental disorder, and these disorders themselves exist on a spectrum of symptom severity that may not always be clinically diagnosed. Since the dynamic nature of epigenetic modifications enables them to be influenced by various intrinsic and environmental factors, it would be unreasonable to expect an absolute effect similar to a genetic mutation. Therefore, we instead predict that a threshold for APA-induced epigenetic alterations exists, and only if surpassed, culminates in a predisposition to disease and ultimately an observed phenotype in offspring. Ongoing investigation into specific genes which consistently show altered methylation patterns will further our understanding of the role of paternal age in the etiology of these neurological conditions.

While our results revealed potential expression markers for susceptibility that can be detected as early as the first blastocyst tissue lineage division, the lack of correlation between the ICM and TE makes transcription profiling of biopsied TE cells futile. Rather, the placenta-brain-axis (PBA) relationship supports the idea that TE cells are a promising avenue for classifying DNA methylation errors at genes that also function in the fetal brain, with possible associations with future neurodevelopmental disorders. In this fashion, we may achieve a way to epigenetically rank embryos for offspring disease risk assessment using biopsied TE cells from IVF blastocysts.

## Conclusions

To summarize, an increased risk for neurodevelopmental disorders, including autism spectrum disorder and schizoprenia, have been observed in children conceived by fathers of advanced paternal age. Our data confirms that as early as the preimplantation embryonic stage, the ICM of embryos derived from APA fathers display transcriptomic dysregulation involving numerous neuronal signaling pathways. Meanwhile, no significant pathway signaling disruption was observed in trophectoderm cells, consistent with clinical findings where APA-derived embryos are not typically compromised during the pre- and post-implantation stages. Instead, both the ICM and TE lineages display methylome changes involving neurotransmission and neuronal signaling, highly enriched for genes implicated in neurodevelopmental disorders including autism spectrum disorder and schizophrenia, and numerous imprinted genes. These genes may have an increased susceptibility to epimutations in the sperm as men age, are likely inherited via fertilization with the potential to persist throughout embryonic development and after birth. Thus, disease risk may be identifiable as early as the first cell lineage differentiation during preimplantation embryonic development, and following further investigations for threshold and cumulative risk of specific target genes, trophectoderm DNA methylation may be a future avenue for epigenetic embryo ranking in couples with advanced paternal age.

### Electronic supplementary material

Below is the link to the electronic supplementary material.

Supplementary Material 1

Supplementary Material 2

Supplementary Material 3

Supplementary Material 4

Supplementary Material 5

Supplementary Material 6

Supplementary Material 7

Supplementary Material 8

Supplementary Material 9

Supplementary Material 10

Supplementary Material 11

## Data Availability

The datasets used and analyzed during the current study are available from the corresponding author on reasonable request.

## References

[1] Tiegs AW, Sachdev NM, Grifo JA, McCulloh DH, Licciardi F (2017). Paternal age is not Associated with pregnancy outcomes after single thawed euploid blastocyst transfer. Reprod Sci.

[2] Sharma R, Agarwal A, Rohra VK, Assidi M, Abu-Elmagd M, Turki RF (2015). Effects of increased paternal age on sperm quality, reproductive outcome and associated epigenetic risks to offspring. Reprod Biol Endocrinol.

[3] Begueria R, Garcia D, Obradors A, Poisot F, Vassena R, Vernaeve V (2014). Paternal age and assisted reproductive outcomes in ICSI donor oocytes: is there an effect of older fathers?. Hum Reprod.

[4] Chapuis A, Gala A, Ferrieres-Hoa A, Mullet T, Bringer-Deutsch S, Vintejoux E (2017). Sperm quality and paternal age: effect on blastocyst formation and pregnancy rates. Basic Clin Androl.

[5] Sagi-Dain L, Sagi S, Dirnfeld M (2016). The Effect of Paternal Age on Oocyte Donation outcomes. Obstet Gynecol Surv.

[6] Hanson BM, Kim JG, Osman EK, Tiegs AW, Lathi RB, Cheng PJ (2020). Impact of paternal age on embryology and pregnancy outcomes in the setting of a euploid single-embryo transfer with ejaculated sperm: retrospective cohort study. F S Rep.

[7] Frattarelli JL, Miller KA, Miller BT, Elkind-Hirsch K, Scott RT (2008). Jr. Male age negatively impacts embryo development and reproductive outcome in donor oocyte assisted reproductive technology cycles. Fertil Steril.

[8] Robertshaw I, Khoury J, Abdallah ME, Warikoo P, Hofmann GE (2014). The effect of paternal age on outcome in assisted reproductive technology using the ovum donation model. Reprod Sci.

[9] Lian ZH, Zack MM, Erickson JD (1986). Paternal age and the occurrence of birth defects. Am J Hum Genet.

[10] Olshan AF, Schnitzer PG, Baird PA (1994). Paternal age and the risk of congenital heart defects. Teratology.

[11] McIntosh GC, Olshan AF, Baird PA (1995). Paternal age and the risk of birth defects in offspring. Epidemiology.

[12] Perrin MC, Brown AS, Malaspina D (2007). Aberrant epigenetic regulation could explain the relationship of paternal age to schizophrenia. Schizophr Bull.

[13] Oksuzyan S, Crespi CM, Cockburn M, Mezei G, Kheifets L (2012). Birth weight and other perinatal characteristics and childhood leukemia in California. Cancer Epidemiol.

[14] Murray L, McCarron P, Bailie K, Middleton R, Davey Smith G, Dempsey S (2002). Association of early life factors and acute lymphoblastic leukaemia in childhood: historical cohort study. Br J Cancer.

[15] Hemminki K, Kyyronen P, Vaittinen P (1999). Parental age as a risk factor of childhood leukemia and brain cancer in offspring. Epidemiology.

[16] Yip BH, Pawitan Y, Czene K (2006). Parental age and risk of childhood cancers: a population-based cohort study from Sweden. Int J Epidemiol.

[17] Janeczko D, Holowczuk M, Orzel A, Klatka B, Semczuk A (2020). Paternal age is affected by genetic abnormalities, perinatal complications and mental health of the offspring. Biomed Rep.

[18] Croen LA, Najjar DV, Fireman B, Grether JK (2007). Maternal and paternal age and risk of autism spectrum disorders. Arch Pediatr Adolesc Med.

[19] Frans EM, Sandin S, Reichenberg A, Lichtenstein P, Langstrom N, Hultman CM (2008). Advancing paternal age and bipolar disorder. Arch Gen Psychiatry.

[20] Malaspina D, Corcoran C, Fahim C, Berman A, Harkavy-Friedman J, Yale S (2002). Paternal age and sporadic schizophrenia: evidence for de novo mutations. Am J Med Genet.

[21] Hare EH, Moran PA (1979). Raised parental age in psychiatric patients: evidence for the constitutional hypothesis. Br J Psychiatry.

[22] Miller B, Messias E, Miettunen J, Alaraisanen A, Jarvelin MR, Koponen H (2011). Meta-analysis of paternal age and schizophrenia risk in male versus female offspring. Schizophr Bull.

[23] Reik W, Dean W, Walter J (2001). Epigenetic reprogramming in mammalian development. Science.

[24] Kobayashi H, Sakurai T, Imai M, Takahashi N, Fukuda A, Yayoi O (2012). Contribution of intragenic DNA methylation in mouse gametic DNA methylomes to establish oocyte-specific heritable marks. PLoS Genet.

[25] Denomme MM, Haywood ME, Parks JC, Schoolcraft WB, Katz-Jaffe MG (2020). The inherited methylome landscape is directly altered with paternal aging and associated with offspring neurodevelopmental disorders. Aging Cell.

[26] Gardner DK, Schoolcraft WB. In vitro culture of human blastocysts. R Jansen, & D Mortimer, editors Towards reproductive certainty: Fertility and genetics beyond. 1999:(pp. 378–88).

[27] Schoolcraft WB, Katz-Jaffe MG (2013). Comprehensive chromosome screening of trophectoderm with vitrification facilitates elective single-embryo transfer for infertile women with advanced maternal age. Fertil Steril.

[28] Raine A, Lundmark A, Annett A, Wiman AC, Cavalli M, Wadelius C (2022). scSPLAT, a scalable plate-based protocol for single cell WGBS library preparation. Sci Rep.

[29] Trubetskoy V, Pardinas AF, Qi T, Panagiotaropoulou G, Awasthi S, Bigdeli TB (2022). Mapping genomic loci implicates genes and synaptic biology in schizophrenia. Nature.

[30] Cooper TG, Noonan E, von Eckardstein S, Auger J, Baker HW, Behre HM (2010). World Health Organization reference values for human semen characteristics. Hum Reprod Update.

[31] Jenkins TG, Aston KI, Pflueger C, Cairns BR, Carrell DT (2014). Age-associated sperm DNA methylation alterations: possible implications in offspring disease susceptibility. PLoS Genet.

[32] Cao M, Shao X, Chan P, Cheung W, Kwan T, Pastinen T (2020). High-resolution analyses of human sperm dynamic methylome reveal thousands of novel age-related epigenetic alterations. Clin Epigenetics.

[33] de Kluiver H, Buizer-Voskamp JE, Dolan CV, Boomsma DI (2017). Paternal age and psychiatric disorders: a review. Am J Med Genet B Neuropsychiatr Genet.

[34] Oluwayiose OA, Wu H, Saddiki H, Whitcomb BW, Balzer LB, Brandon N (2021). Sperm DNA methylation mediates the association of male age on reproductive outcomes among couples undergoing infertility treatment. Sci Rep.

[35] Milekic MH, Xin Y, O’Donnell A, Kumar KK, Bradley-Moore M, Malaspina D (2015). Age-related sperm DNA methylation changes are transmitted to offspring and associated with abnormal behavior and dysregulated gene expression. Mol Psychiatry.

[36] Kidd SA, Eskenazi B, Wyrobek AJ (2001). Effects of male age on semen quality and fertility: a review of the literature. Fertil Steril.

[37] Guibert S, Forne T, Weber M (2012). Global profiling of DNA methylation erasure in mouse primordial germ cells. Genome Res.

[38] Hajkova P, Erhardt S, Lane N, Haaf T, El-Maarri O, Reik W (2002). Epigenetic reprogramming in mouse primordial germ cells. Mech Dev.

[39] Popp C, Dean W, Feng S, Cokus SJ, Andrews S, Pellegrini M (2010). Genome-wide erasure of DNA methylation in mouse primordial germ cells is affected by AID deficiency. Nature.

[40] Jaffe AB, Hall A (2005). Rho GTPases: biochemistry and biology. Annu Rev Cell Dev Biol.

[41] Tremblay MW, Jiang YH (2019). DNA methylation and susceptibility to Autism Spectrum Disorder. Annu Rev Med.

[42] Magwai T, Shangase KB, Oginga FO, Chiliza B, Mpofana T, Xulu KR (2021). DNA methylation and Schizophrenia: current literature and future perspective. Cells.

[43] Nardone S, Sams DS, Reuveni E, Getselter D, Oron O, Karpuj M (2014). DNA methylation analysis of the autistic brain reveals multiple dysregulated biological pathways. Transl Psychiatry.

[44] Denomme MM, Haywood ME, McCallie BR, Schoolcraft WB, Katz-Jaffe MG (2021). The prolonged disease state of infertility is associated with embryonic epigenetic dysregulation. Fertil Steril.

[45] Crespi B (2008). Genomic imprinting in the development and evolution of psychotic spectrum conditions. Biol Rev Camb Philos Soc.

[46] Smith RG, Reichenberg A, Kember RL, Buxbaum JD, Schalkwyk LC, Fernandes C (2013). Advanced paternal age is associated with altered DNA methylation at brain-expressed imprinted loci in inbred mice: implications for neuropsychiatric disease. Mol Psychiatry.

[47] Rasmussen AH, Rasmussen HB, Silahtaroglu A (2017). The DLGAP family: neuronal expression, function and role in brain disorders. Mol Brain.

[48] Soler J, Fananas L, Parellada M, Krebs MO, Rouleau GA, Fatjo-Vilas M (2018). Genetic variability in scaffolding proteins and risk for schizophrenia and autism-spectrum disorders: a systematic review. J Psychiatry Neurosci.

[49] Denomme MM, Parks JC, McCallie BR, McCubbin NI, Schoolcraft WB, Katz-Jaffe MG (2020). Advanced paternal age directly impacts mouse embryonic placental imprinting. PLoS ONE.

[50] Choufani S, Turinsky AL, Melamed N, Greenblatt E, Brudno M, Berard A (2019). Impact of assisted reproduction, infertility, sex and paternal factors on the placental DNA methylome. Hum Mol Genet.

[51] Rosenfeld CS (2021). The placenta-brain-axis. J Neurosci Res.

[52] Bahado-Singh RO, Vishweswaraiah S, Aydas B, Radhakrishna U (2021). Placental DNA methylation changes and the early prediction of autism in full-term newborns. PLoS ONE.

[53] Ravaei A, Emanuele M, Nazzaro G, Fadiga L, Rubini M (2023). Placental DNA methylation profile as predicting marker for autism spectrum disorder (ASD). Mol Med.

[54] Laufer BI, Neier K, Valenzuela AE, Yasui DH, Schmidt RJ, Lein PJ (2022). Placenta and fetal brain share a neurodevelopmental disorder DNA methylation profile in a mouse model of prenatal PCB exposure. Cell Rep.

[55] Zhu Y, Zhang Y, Jin Y, Jin H, Huang K, Tong J (2023). Identification and prediction model of placenta-brain axis genes associated with neurodevelopmental delay in moderate and late preterm children. BMC Med.

[56] Dong W, Todd AC, Broer A, Hulme SR, Broer S, Billups B (2018). PKC-Mediated modulation of astrocyte SNAT3 glutamine transporter function at synapses in situ. Int J Mol Sci.

[57] Kim H, Lee Y, Park JY, Kim JE, Kim TK, Choi J (2017). Loss of Adenylyl Cyclase Type-5 in the dorsal striatum produces autistic-like behaviors. Mol Neurobiol.

[58] Dawson VL, Dawson TM (1998). Nitric oxide in neurodegeneration. Prog Brain Res.

[59] Quitterer U, AbdAlla S (2020). Improvements of symptoms of Alzheimer;s disease by inhibition of the angiotensin system. Pharmacol Res.

[60] Tilot AK, Kucera KS, Vino A, Asher JE, Baron-Cohen S, Fisher SE (2018). Rare variants in axonogenesis genes connect three families with sound-color synesthesia. Proc Natl Acad Sci U S A.

[61] Anitha A, Nakamura K, Thanseem I, Yamada K, Iwayama Y, Toyota T (2012). Brain region-specific altered expression and association of mitochondria-related genes in autism. Mol Autism.

[62] Chu TT, Liu Y (2010). An integrated genomic analysis of gene-function correlation on schizophrenia susceptibility genes. J Hum Genet.

[63] Sagi-Dain L, Sagi S, Dirnfeld M (2015). Effect of paternal age on reproductive outcomes in oocyte donation model: a systematic review. Fertil Steril.

[64] Di Maio V (2021). The glutamatergic synapse: a complex machinery for information processing. Cogn Neurodyn.

[65] de Sena Brandine G, Aston KI, Jenkins TG, Smith AD (2023). Global effects of identity and aging on the human sperm methylome. Clin Epigenetics.

