# Supplementary figures and images for: Paternal aging impacts expression and epigenetic markers as early as the first embryonic tissue lineage differentiation
Source: Hum Genomics. 2024 Mar 26;18:32. doi: 10.1186/s40246-024-00599-4 (PMC10964547; doi:10.1186/s40246-024-00599-4)

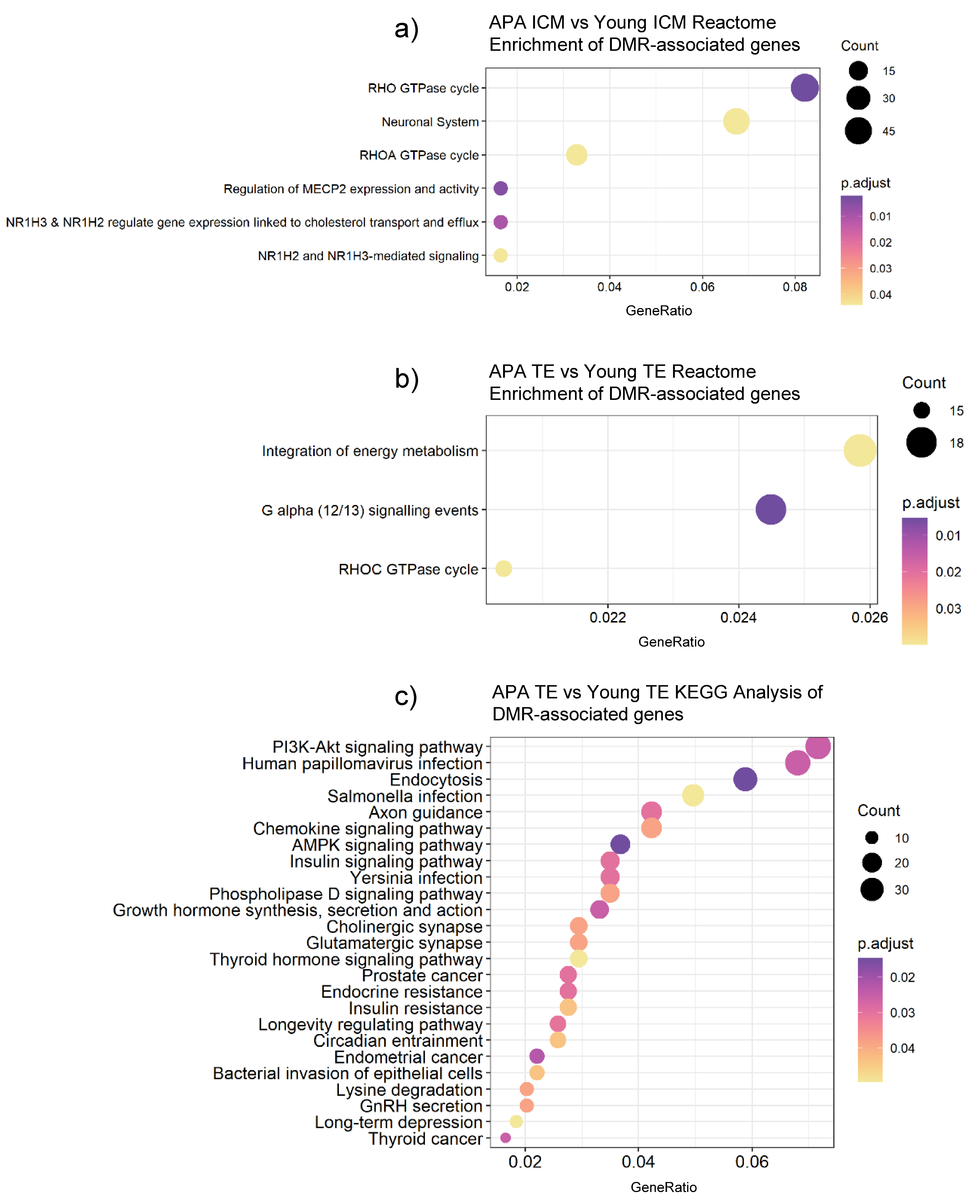

Supplement: Supplementary file 1 — Supplementary Material 1 [file 40246_2024_599_MOESM1_ESM.tif]

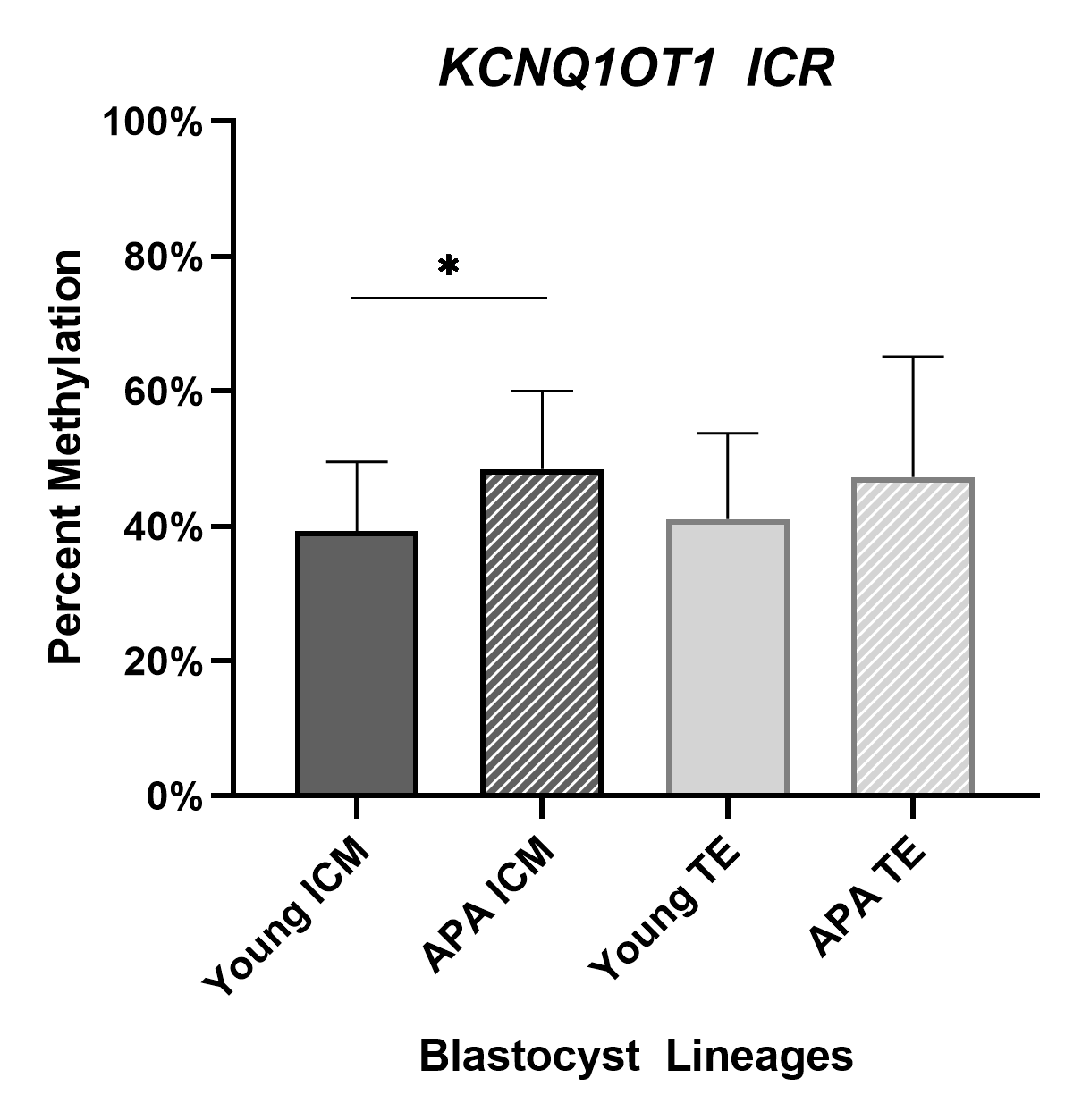

Supplement: Supplementary file 2 — Supplementary Material 2 [file 40246_2024_599_MOESM2_ESM.tif]

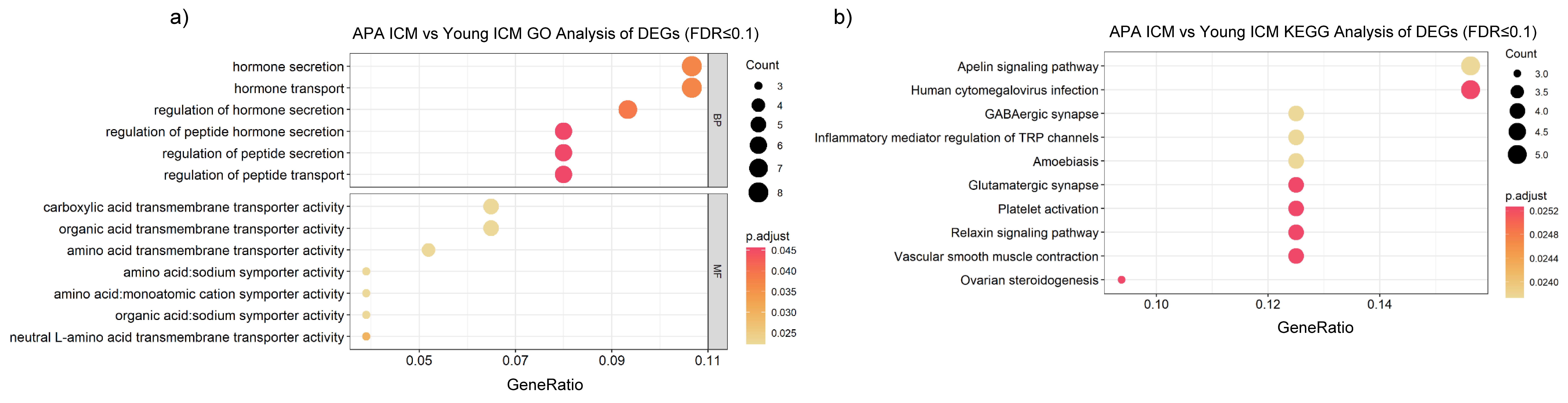

Supplement: Supplementary file 3 — Supplementary Material 3 [file 40246_2024_599_MOESM3_ESM.tif]

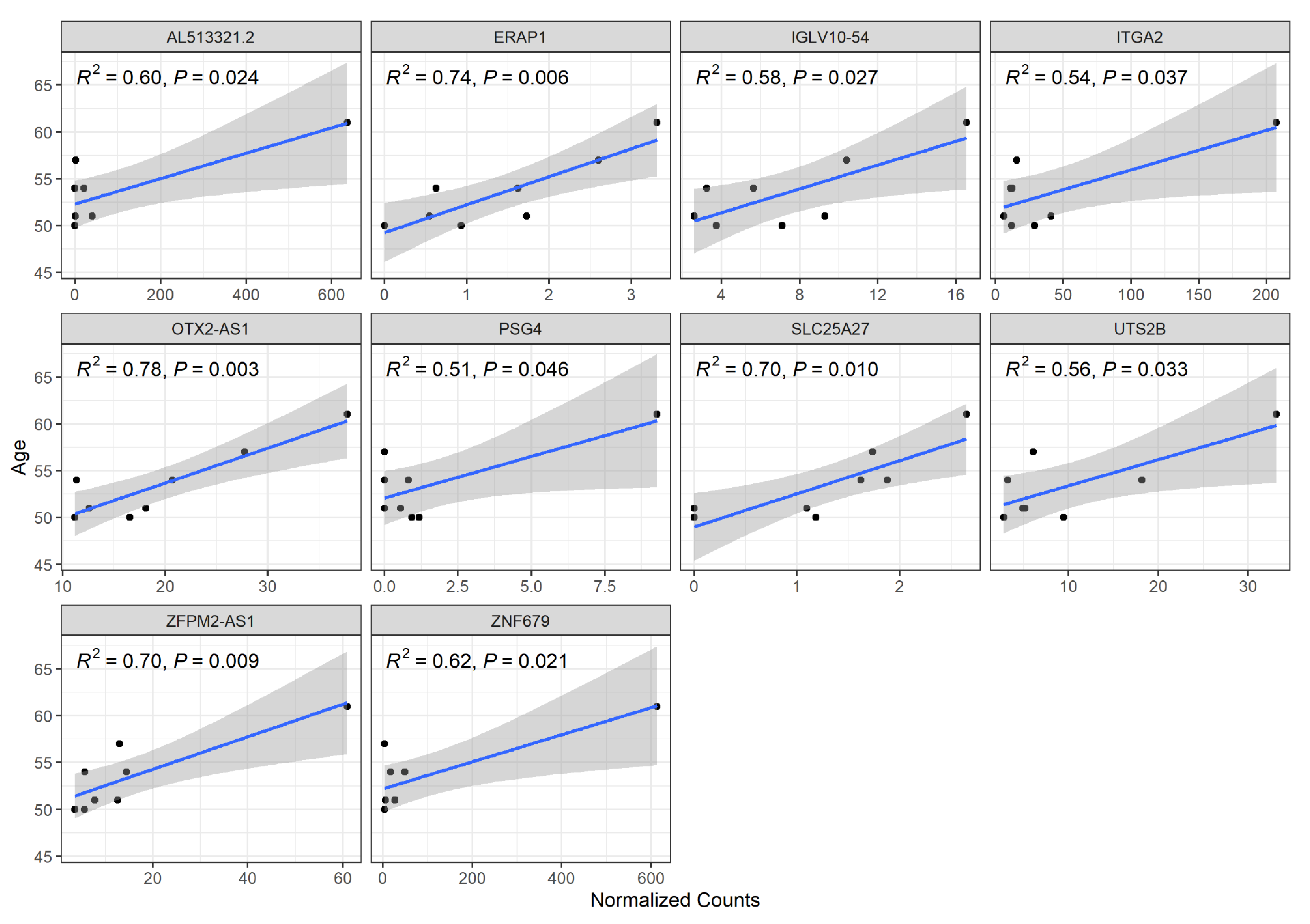

Supplement: Supplementary file 4 — Supplementary Material 4 [file 40246_2024_599_MOESM4_ESM.tif]
